# Rhinitis, sinusitis and ocular disease – 2089. Evaluating the quality and contents in paranasal sinusitis-related information on the web-sites in western physicians and eastern physicians

**DOI:** 10.1186/1939-4551-6-S1-P168

**Published:** 2013-04-23

**Authors:** Yong-Dae Kim, Si-Youn Song, Chang Hoon Bae, Heung-Man Lee

**Affiliations:** 1 Department of Otorhinolaryngology-Head and Neck Surgery, College of Medicine, Yeungnam University, Daegu, South Korea; 2 Otorhinolaryngology-Head and Neck Surgery, Korea University College of Medicine, Guro Hospital, Seoul, South Korea

## Background

A large amount of information related to paranasal sinusitis is available on the internet; however, little is known about the quality and content of such information. We assessed paranasal sinusitis-related information on web sites of western and eastern physicians.

## Methods

Using the search engine of Naver and Daum, a total of 159 web sites (108 by western physicians, 51 by eastern physicians) were identified by the keyword search “paranasal sinusitis” and were classified using the categories of location and field of contents. These web sites were evaluated for several aspects, and an informational value score was assigned to each.

## Results

Regarding the location, Seoul (38), Gyeonggi (28), Busan (10) accounted for 70% of sites by western physicians, compared with 76% for the eastern physicians [Seoul (27), Gyeonggi (9), Busan (3)]. With respect to content, main content and Q/A were excellent in sites by eastern physicians whereas clinical schedule was excellent in those by western physicians. The mean informational value of the web sites was 5.17 for western physicians and 2.19 for eastern physicians (p<0.05).

## Conclusions

Web sites on paranasal sinusitis concentrated very firmly on metropolitan areas. Web sites by western physicians provided better paranasal sinusitis-related information than those by eastern physicians.

